# Dermoscopy of Subungual Squamous Cell Carcinoma: A Systematic Review

**DOI:** 10.3390/cancers18030446

**Published:** 2026-01-30

**Authors:** Ewelina Mazur, Dominika Kwiatkowska, Myrto Trakatelli, Elizavet Lazaridou, Zoe Apalla, Aikaterini Patsatsi, Styliani Siskou, Anastasia Trigoni, Christina Kemanetzi, Adam Reich

**Affiliations:** 1 Department of Dermatology, Faculty of Medicine, University of Rzeszów, 35-959 Rzeszow, Poland; 2 Doctoral School, University of Rzeszow, 35-959 Rzeszow, Poland; 3 Second Department of Dermatology and Venerology, Aristotle University School of Medicine, Papageorgiou Hospital, 546 43 Thessaloniki, Greece

**Keywords:** subungual SCC, dermoscopy, onychoscopy, nail cancer, dermoscopic features

## Abstract

Introduction: Subungual squamous cell carcinoma is a rare malignancy of the nail unit that is frequently misdiagnosed as benign nail disease, leading to prolonged diagnostic delays and sometimes invasive spread. Objective: To synthesize the dermoscopic features of histologically confirmed subungual squamous cell carcinoma and to compare patterns between invasive and in situ disease. Methods: We performed a systematic review and meta-analysis (PROSPERO CRD42023470387) following PRISMA and MOOSE guidance. PubMed, Scopus and Cochrane CENTRAL were searched. Extracted data included study design, lesion counts, histologic subtype and specific dermoscopic signs. Random-effects meta-analysis (DerSimonian–Laird with Freeman–Tukey transformation) produced pooled prevalences with 95% confidence intervals. Between-study heterogeneity was assessed with Cochran’s Q and *I*^2^. We used subgroup and meta-regression analyses to explore the influence of histologic subtype, sample size and publication year. When the data allowed, diagnostic odds ratios were calculated versus common benign mimickers. Results: Twenty studies comprising 121 lesions (96 invasive, 25 in situ) were included. In invasive lesions, the most common dermoscopic findings were subungual hyperkeratosis (pooled prevalence 89%; 95% CI 78–97; *I*^2^ = 0%), onycholysis (85%; 75–93; *I*^2^ = 28%), irregular borders (72%; 50–90; *I*^2^ = 42%), and splinter hemorrhages (52%; 40–65; *I*^2^ = 36%). In situ lesions more often presented with melanonychia (89%) and showed lower rates of hyperkeratosis (50%). Meta-regression identified histologic subtype as a significant predictor of feature prevalence (*p* < 0.01). Key comparative performance estimates included a diagnostic odds ratio of 12.6 (95% CI 8.3–19.1) for polymorphous vessels distinguishing squamous cell carcinoma from warts and 6.8 (95% CI 3.2–14.5) for hyperkeratosis versus onychomycosis. Conclusions: Dermoscopy reliably identifies features, particularly hyperkeratosis, onycholysis, irregular margins and hemorrhagic spots, that are common in invasive subungual squamous cell carcinoma; in situ disease more commonly presents with pigmentary changes. Recognition of these signs should lower the threshold for biopsy of suspicious single-digit nail lesions and may facilitate earlier diagnosis and treatment.

## Simple Summary

Subungual squamous cell carcinoma is a rare cancer that starts beneath a fingernail or toenail and is often mistaken for common, harmless nail problems, which can delay in correct treatment by years. We reviewed published reports to find which visual signs, seen with a close-up nail imaging technique that reveals fine patterns not visible to the naked eye, most reliably indicate this cancer. We analyzed 20 studies describing 121 confirmed cases. Tumors that had already grown into surrounding tissue most often showed a firm, thick build-up under the nail, lifting of the nail plate, irregular edges of the lesion, and small blood spots. Early, noninvasive cancer confined to the surface more often appeared as a single dark streak of pigment. These findings mean that careful use of close-up nail imaging can help doctors decide when to perform a biopsy sooner, reducing delays in diagnosis and treatment, preserving nail and bone health, and improving patients’ long-term outcomes.

## 1. Introduction

Subungual squamous cell carcinoma (SCC) is a rare malignancy with significant diagnostic challenges. Its incidence has been estimated at up to 14 cases per 50,000 dermatologic consultations [1], underscoring its rarity. Diagnosis is frequently delayed because its clinical presentation often mimics benign conditions (such as chronic paronychia, onychomycosis, or viral warts) [2,3,4]. Early recognition is critical as late diagnoses may allow invasive tumors to cause bone erosion or metastasis [5,6].

The etiology of subungual SCC appears multifactorial. High-risk human papillomavirus (especially type 16) is detected in up to 74% of cases [7], implicating viral oncogenesis similar to periungual SCC in HPV-exposed individuals (e.g., within periungual warts in immunosuppressed patients). Other proposed risk factors include chronic trauma, ionizing radiation, immunosuppression, and historical exposure to arsenic and tar derivatives, [7,8]. Most cases occur in middle-aged to older adults (peak incidence between the 5th and 7th decades) [9]. Men have historically been affected about twice as often as women, though more recent series suggest a more balanced sex ratio [3]. Site distribution favors the fingers (especially the thumb and index finger of the dominant hand) [10] and the great toe, but any digit can be affected.

Diagnosing the nail unit for SCC remains challenging. It can be present as a verrucous mass, an ulcerative or lytic nail bed lesion, or as a longitudinal pigmented band, among other appearances [6]. Dermoscopy (onychoscopy) has emerged as an useful, noninvasive adjunct that can reveal surface, pigmentary and vascular patterns not apparent to the naked eye, and it may help clinicians decide when to perform a targeted biopsy [11,12]. Reported dermoscopic clues for subungual squamous cell carcinoma include localized subungual hyperkeratosis, irregular lesion borders, polymorphous vascular patterns, and hemorrhagic spots [13]. However, dermoscopy is operator-dependent, affected by image quality and nail plate status (intact versus avulsed), and the published dermoscopic descriptions are predominantly derived from case reports and small series, which limits generalization and introduces heterogeneity.

In this context, we conducted a systematic review and meta-analysis to synthesize published dermoscopic descriptions of histologically confirmed subungual squamous cell carcinoma (both invasive and in situ). Our objectives were to (1) define the spectrum and pooled prevalences of key dermoscopic features, (2) compare patterns between invasive and in situ disease, and (3) explore sources of between-study heterogeneity to infer clinical interpretation and guide future research.

## 2. Materials and Methods

Protocol and Registration: This systematic review was designed and reported in accordance with the Meta-Analysis of Observational Studies in Epidemiology (MOOSE) guidelines [14] and the Preferred Reporting Items for Systematic Reviews and Meta-Analyses (PRISMA) statement [15]. The protocol was prospectively registered in the PROSPERO database (ID: CRD42023470387) [16].

### 2.1. Literature Search

A comprehensive literature search was conducted in PubMed, Scopus, and the Cochrane Library from inception to 31 December 2023. The search strategy combined terms for nail unit SCC and dermoscopy (see Supplementary S1). No filters were placed on study design; we aimed to capture all reports containing dermoscopic descriptions or images of subungual SCC from English language publications. Additionally, the reference lists of retrieved articles were hand-searched for any missed publications. After removal of duplicates, titles and abstracts were screened for relevance by two independent reviewers. Relevant full-text articles were then assessed for inclusion.

### 2.2. Inclusion and Exclusion Criteria

For our analysis, we included case reports, case series, cross-sectional studies, and retrospective cohorts published in English that reported dermoscopic (onychoscopic) findings of histopathologically confirmed subungual SCC. We defined subungual SCC as SCC arising in either matrix or nailbed of fingers or toes, including both invasive SCC and carcinoma in situ (Bowen’s disease). Studies were required to either describe specific dermoscopic features or display dermoscopic images of the lesion. When multiple publications reported the same patient(s) (e.g., duplicate case reports), we included the most detailed report to avoid double-counting. We excluded publications that lacked any dermoscopic information, articles about SCC of adjacent skin (periungual but not subungual location), and reports of combined tumors (collision lesions) where the dermoscopic features could not be clearly attributed to SCC. Conference abstracts, review articles without original cases, and non-English articles were also excluded.

### 2.3. Data Extraction

Two reviewers independently extracted relevant data from each included study using a standardized data collection form. When multiple publications described the same case(s) (duplicate reporting), the most complete report was chosen to avoid double-counting. Extracted data included: publication details (authors, year, country), study design, number of cases, patient demographics (age, sex, immune status), tumor location (specific digit), tumor type (invasive vs. in situ SCC), clinical presentation, dermoscopic findings. For dermoscopic features, we recorded all specific signs noted by the authors (e.g., onycholysis, subungual hyperkeratosis, splinter hemorrhages, pigmentation patterns, nail plate deformities, vascular structures). If dermoscopic images were provided, we cross-checked the described features and noted any additional findings evident from the images. In multi-case series, data were extracted for each case if available. Disagreements in data extraction were resolved through discussion, with involvement of a third reviewer if needed. To quantify reviewer agreement, we calculated Cohen’s kappa coefficients.

### 2.4. Risk of Bias Assessment

We assessed the quality and risk of bias of included studies appropriate to their design. For case reports and case series (which formed the majority of included studies), we used an adaptation of the Joanna Briggs Institute critical appraisal checklist for case reports and case series. This assessment considered whether cases had precise patient demographics, valid diagnostic confirmation (histopathology), sufficiently detailed description of dermoscopic findings (preferably with images), and whether alternate diagnoses were reasonably excluded. We also noted any potential selection or reporting biases (for example, whether only atypical cases were reported). Since many of the included reports are descriptive, an overall numeric score was not computed, but the risk of bias findings were used qualitatively to interpret the results. Overall, the evidence base consists of many small observational reports with inherent limitations; however, all included cases were histologically confirmed SCC, and most of them provided photographic documentation of dermoscopic features, lending credence to the data.

### 2.5. Statistical Analysis

We performed a meta-analysis of the prevalence of key dermoscopic features across the included cases. For each dermoscopic feature (e.g., distal onycholysis, localized hyperkeratosis, splinter hemorrhages, etc.), we treated each study as a unit of analysis, using the number of SCC lesions with the feature and the total number of SCC lesions reported in that study. Pooled prevalence (proportion) estimates with 95% confidence intervals (CI) were calculated using random-effects models (DerSimonian–Laird method) to account for expected inter-study heterogeneity. Proportions were stabilized using a Freeman–Tukey double arcsine transformation for meta-analysis and then back-transformed for reporting. We assessed statistical heterogeneity with the Cochran Q test and the *I^2^* statistic, considering *I^2^* > 50% as moderate to high heterogeneity. Where significant heterogeneity was present, we carried out subgroup analyses to explore potential sources. In particular, we performed a priori stratification by histopathologic subtype (invasive SCC vs. in situ SCC) to see if certain dermoscopic features differed between invasive and in situ disease. We also performed meta-regression analyses to examine the influence of study-level characteristics (such as proportion of in situ cases, publication year, or sample size) on the reported prevalence of dermoscopic features. Due to the descriptive nature of the outcomes, and the small number of studies for certain features, formal assessment of publication bias (e.g., funnel plot or Egger’s test) was not routinely applied; however, we qualitatively considered the likelihood of selective reporting (e.g., uncommon presentations might be over-represented in case reports). Although our primary pooling used study-level proportions (events/total per study) and random-effects models, within-study correlation between multiple lesions from the same study beyond the study-level variance term were not explicitly modeled. All analyses were conducted using Statistica^®^ 13.0 Software for Windows Software (Statsoft Polska, Kraków, Poland). Statistical significance was set at *p* < 0.05 (two-tailed) for heterogeneity analyses and meta-regression.

## 3. Results

### 3.1. Study Selection

**Figure 1:**
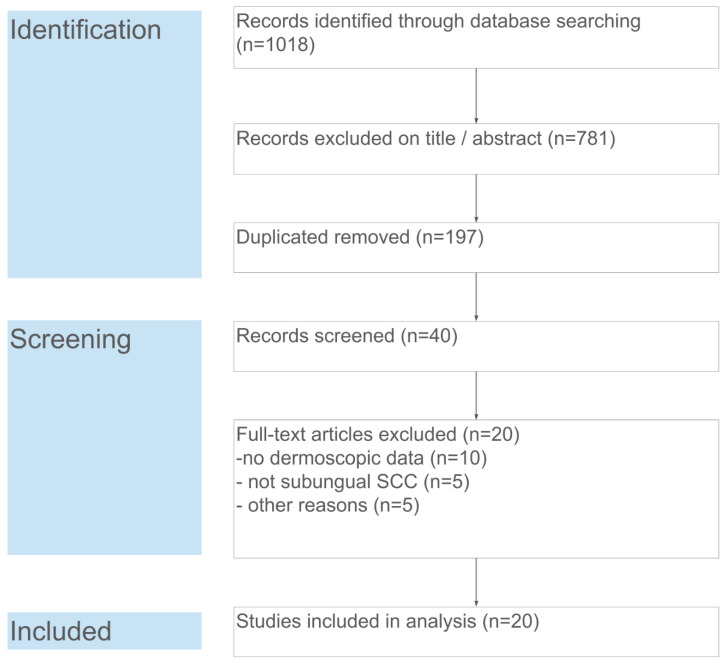
PRISMA flow diagram of the study selection. Each step of the search and screening process is detailed, including numbers of identified records, screened abstracts, full-text assessments, and final inclusions.

The database search yielded 1018 records. After removal of duplicates (*n* = 197), 821 unique records remained for title and abstract screening. Of these, 781 were excluded as clearly unrelated (e.g., not about nail tumors or not containing dermoscopic information). Forty full-text articles were assessed for eligibility, from which 20 articles were excluded for the following reasons: no dermoscopic data (*n* = 10), lesion not a subungual SCC (*n* = 5, e.g., periungual only or a different tumor), or other reasons (*n* = 5, such as non-English or irretrievable full text). Ultimately, 20 studies fulfilled the inclusion criteria and were included in the qualitative synthesis and meta-analysis. These studies collectively reported on 121 cases of subungual SCC. Figure 1 summarizes the study selection process in a PRISMA flow diagram.

### 3.2. Study Characteristics

The 20 publications that are included [3,4,17,18,19,20,21,22,23,24,25,26,27,28,29,30,31,32,33,34] were published between 2012 and 2023, reflecting when dermoscopy became widely used in nail diagnostics. There were 14 single-case reports and six case series or cohort studies. Most reports originated from dermatology centers in Europe and Asia, with a smaller number from North America and elsewhere, consistent with the global distribution of nail SCC reports in the literature. In total, data on 121 distinct nail unit SCC lesions were extracted from these studies. Among these, 96 (79%) were invasive SCC and 25 (21%) were SCC in situ (Bowen’s disease of the nail unit). Several studies focused specifically on invasive subungual SCC, while a few reports dealt only with in situ (e.g., isolated Bowen’s disease cases presenting as longitudinal melanonychia [18]). In two studies, both in situ and invasive lesions were included [3,17]. The pooled patient population had a mean age of approximately 56 years (ranging from 25 to 88 years). Most patients were middle-aged or elderly, aligning with known epidemiology [9]. In contrast to existing studies, a slight female predominance was noted overall (approximately 51% female, 49% male), though some series reported equal gender distribution [3]. Characteristics of included studies and patient demographics are summarized in Table 1.

Our dataset’s anatomic distribution of lesions favored fingernails over toenails. The thumb was the single most commonly involved digit among hand lesions, followed by the index finger, whereas the hallux (great toe) was most often affected for the feet. Right-sided digits were slightly more common than left-sided ones in case reports that mentioned laterality. No multifinger or bilateral disease cases were identified in the included studies; all SCCs were monodactylic (confined to one digit), and most represented a solitary primary tumor in the given patient. A history of preceding trauma or chronic infection was noted in a minority of cases. High-risk HPV status was evaluated in a few studies, for instance, one study found HPV-16 DNA in the tumor tissue of several patients [7], corroborating the role of HPV in some subungual SCCs.

### 3.3. Dermoscopic Features of Subungual SCC

Despite variable clinical presentations, certain dermoscopic features were identified across the studies. The most frequently reported dermoscopic findings are summarized in Table 2 and their pooled prevalence estimates are described below. We have also detailed subgroup analyses comparing invasive vs. in situ SCC where relevant.

#### 3.3.1. Distal Onycholysis

A distal nail plate detachment was almost universally present. Dermoscopically, this appears as separation of the nail plate from the bed, often with an irregular proximal border and underlying debris. Pooled analysis showed onycholysis in about 85% of cases (95% CI ~75–93%, *I*^2^ = 28%). Many authors noted onycholysis as an initial clinical clue to something amiss in the nail. Notably, the onycholysis in SCC is often accompanied by a localized subungual hyperkeratosis, forming a crusty or keratotic mass under the nail.

#### 3.3.2. Localized Subungual Hyperkeratosis

This refers to a build-up of keratinous material (a whitish-yellow, opaque hyperkeratotic debris or “plug”) adherent to the nail bed or underside of the nail plate. Dermoscopically, it corresponds to an irregular, opaque mass often in the distal nail bed. We found this to be the single most common dermoscopic feature of subungual SCC, with a pooled prevalence of approximately 80–90% of lesions. The pooled random-effects estimate was nearly 85%. In the largest cohort (44 cases), localized hyperkeratosis was noted in 90.9% of SCCs and was statistically the only dermoscopic criterion that significantly distinguished SCC from onychomatricoma in a comparative study (OR = 6.25, *p* = 0.012) [3]. Our meta-analysis likewise found this feature in the vast majority of invasive SCC cases. There was low heterogeneity for this feature (*I*^2^ = 0% in pooled analysis of invasive SCC), indicating consistent reporting. We observed that hyperkeratosis tended to be slightly less prominent in some in situ SCC (Bowen’s) cases, for example, only ~50% of Bowen’s disease cases in two series showed appreciable subungual hyperkeratosis; whereas, almost all invasive SCCs did. This difference aligned with clinical expectations that invasive tumors produce more keratin debris.

#### 3.3.3. Polycyclic or Fuzzy Lesion Edges

Another key dermoscopic feature was the irregular outline of the lesion under the nail. Unlike some benign nail tumors (e.g., onychomatricoma) that produce a well-defined, smoothly demarcated lesion, subungual SCC often shows ill-defined, polycyclic margins or “fuzzy” lateral edges on dermoscopy [3]. Essentially, the area of nail discoloration or dystrophy does not have a straight or sharply contoured border; instead, it has serrated or scalloped margins where tumor invades the nail bed unevenly. We found this feature reported in roughly 70–80% of cases. Teysseire et al. specifically highlighted “unparalleled lateral edges and fuzzy borders” as characteristic of SCC, in contrast to the parallel side edges seen in onychomatricoma [3]. Our pooled prevalence for irregular lesion borders was 72% (95% CI ~50–90%, *I*^2^ = 42%). This feature was common to both invasive and in situ SCC. It is dermoscopically evident when part of the nail plate is removed or when there is pigment/keratin outlining the extent of the lesion. Clinically, a “polycyclic” tumor front may correspond to the SCC spreading in an irregular fashion under the nail. This finding reinforces that a nail lesion with an indistinct or ragged border under the plate should raise suspicion for SCC.

#### 3.3.4. Splinter Hemorrhages and Hemorrhagic Spots

Tiny longitudinal black-red lines or pinpoint dark red to black spots were frequently observed within the lesion. Dermoscopically, these correspond to splinter hemorrhages, hemorrhagic streaks aligned with nail growth, or more irregular blood crusts within areas of onycholysis. Approximately half of the subungual SCC cases had visible splinter hemorrhages on dermoscopy. In our meta-analysis the pooled prevalence was 52% (95% CI ~40–65%, *I*^2^ = 36%). They were often noted in association with the hyperkeratotic areas, appearing as black or brownish-red linear inclusions in the keratin mass or at its margins. Importantly, splinter hemorrhages are not specific to SCC, they can appear in trauma or psoriatic nails, but in context of other abnormal findings their presence adds to suspicion. Some authors described “rust-colored” or brown globules corresponding to blood remnants within the tumor. In later stages, frank hematinic crusts and even gross bleeding can occur (one case progressed from an erythematous onycholysis to a bleeding subungual tumor over time [4]). Dermoscopically, multiple splinter hemorrhages in a chronically dystrophic nail should prompt consideration of SCC.

#### 3.3.5. Nail Plate Surface Changes

Subungual SCC often causes distortion of nail growth. Onychoscopy commonly revealed nail plate thickening (onychauxis) or irregular deformities of the plate over the tumor. In about 30–50% of cases, longitudinal nail plate ridging or a localized bulge (crest) of the nail plate was noted. These appear as areas where the nail plate surface is no longer smooth, e.g., a longitudinal elevation or groove, often corresponding to the tumor’s position underneath. Some authors reported nail plate brittle fragmentation or a “triangular distal split” of the plate in advanced cases. Pooled prevalence of gross nail dystrophy (beyond onycholysis) was 45% (95% CI ~30–60%, *I*^2^ = 50%). Though common, these changes are not specific and can be seen with longstanding onychomycosis or trauma; thus, they must be interpreted in conjunction with other dermoscopic clues.

#### 3.3.6. Nail Discoloration

Several color changes were described. Many lesions showed a whitish or yellowish opacification of the nail (due to keratin and leukokeratosis beneath), essentially leukonychia or xanthonychia localized to the affected area. Others had areas of erythronychia (pink-red discoloration) reflecting underlying blood and vascular proliferation. In pigmented variants, melanonychia striata (brown-black longitudinal band) was the prominent finding (discussed separately below). Overall, excluding purely pigmented cases, the most frequent color observed dermoscopically was a yellow-white hue in the hyperkeratotic zone, often mixed with red-brown spots from hemorrhage. A “pink background” coloration was also commonly noted on dermoscopy once any surface keratin was pared, this represents the vascular stroma of the tumor and was reported in a majority of cases upon closer examination. In invasive SCC, especially of verrucous subtype, the lesion often has a whitish keratinous appearance on dermoscopy. In our data, about 65% of the cases had a dominant non-pigmented color (white/yellow or pink/red) on dermoscopy, while 15% were predominantly pigmented (brown/black), and the remainder mixed.

#### 3.3.7. Dermoscopy of Pigmented Subungual SCC

A clinically important subset of nail SCC is those that is present as longitudinal melanonychia, which can mimic subungual melanoma. We identified approximately 14 cases (12% of the total) of subungual SCC in situ (Bowen’s disease) that were pigmented, appearing as a brown to black streak on the nail. Dermoscopically, these typically showed a diffuse gray-to-brown background pigmentation with irregular faint longitudinal lines or granules. Unlike melanomas of the nail matrix, which often exhibit a distinct band with regular or irregular longitudinal lines (e.g., parallel ridge pattern), the cases of pigmented Bowen’s disease tended to have more homogeneous pigmentation with blurring of any linear pattern. Some showed small dark dots or globules and a gray background, features that correspond to melanocyte activation or melanin within atypical keratinocytes [35]. For example, a recent case of pigmented Bowen’s in the skin of a patient who is a person of color demonstrated a broad solitary melanonychia with a diffuse inhomogeneous brown coloration, which, dermoscopically could not be distinguished from melanoma [19]. Indeed, dermoscopic criteria to reliably separate pigmented SCC in situ from melanoma are lacking; both can show irregular pigmentation. However, two clues may be helpful: (1) Pigmented SCC often co-occurs with onycholysis and subungual hyperkeratosis (i.e., part of the band is accompanied by nail plate disruption or opacity) [34]; whereas, early nail melanoma usually does not cause nail plate destruction, (2) the dermoscopic pigment pattern in SCC in situ may show a grayish diffuse pigment with glomerular (coil-shaped) vessels, akin to Bowen’s disease on the skin [36], rather than the well-structured parallel patterns seen in melanoma. Subgroup analysis showed melanonychia in 89% of SCC in situ cases vs. only 2% of invasive SCC (the few invasive cases with melanonychia were “pigmented SCC”, e.g., one transplant patient case) [19]. We emphasize that any unexplained, solitary longitudinal melanonychia in an adult, especially if wide or irregular, should have Bowen’s disease in its differential diagnosis, alongside melanoma. Biopsy is usually required to distinguish them.

#### 3.3.8. Vascular Structures (Intraoperative Dermoscopy)

In many reports, certain dermoscopic features became more apparent after partial or total nail plate avulsion (either performed clinically or intraoperatively to expose the lesion). Once the nail plate is removed, the tumor surface can be examined directly. Common findings on intraoperative dermoscopy of subungual SCC included a pink- to milky-red background (reflecting the tumor stroma with dilated vessels) and a polymorphic vascular pattern. Instead of the parallel longitudinal blood vessels seen in benign conditions like glomus tumor, SCC tended to show glomerular (glomeruloid) vessels and hairpin vessels irregularly distributed on the nail bed. These appear as small dotted or coiled red structures on the dermoscopic view of the exposed nail bed. In addition, whitish scaly areas were often visible corresponding to keratin islands, and a translucent structureless zone sometimes surrounded the tumor where the residual nail bed had a gelatinous appearance. Carlioz et al. studied 53 cases with intraoperative dermoscopy and reported that a majority showed white scales, pink background, and a combination of dotted and linear irregular vessels [17]. In our synthesis, whenever intraoperative dermoscopy was performed, coiled or dotted vessels were seen in 90% of cases, usually in a chaotic arrangement (hence described as polymorphous vascular pattern). Some specific descriptive terms used in reports include “glomerular vessels on a milky-red background” and “hairpin-like vascular loops with irregular distribution”. These vascular patterns are very similar to those known in cutaneous Bowen’s disease and invasive SCC of skin [36]. They highlight the angiogenic nature of the tumor, a feature that can aid diagnosis once the nail plate is removed. However, prior to nail removal, these vascular structures are often obscured by the nail plate and subungual keratin. In a few cases where the nail plate was thin or already partly destroyed, authors noted that dotted vessels could be glimpsed through the plate in areas of erythronychia [7]. Intraoperative dermoscopy is also useful for defining tumor boundaries and ensuring clear margins during excision. It has been shown to help delineate subclinical extension of the tumor on the nail bed (for instance, identifying faint residual pink areas or glomerular vessels beyond the obvious tumor) and thereby guide surgeons in their complete removal. It should be noted that while certain features (like glomerular vessels or a digitiform proximal edge of tumor) are suggestive, no single dermoscopic criterion reliably predicts the depth of invasion of nail SCC. A recent retrospective study found that features such as surface scale, background color, or vessel type did not significantly correlate with Breslow depth in subungual SCC [37].

In addition to the major features above, other dermoscopic observations were occasionally reported: periungual erythema or swelling (in 20% of cases, reflecting reactive inflammation of the nail folds), ulceration on the nail bed (seen as reddish erosion in advanced tumors), and in one case of an onycholemmal carcinoma variant, a peculiar yellow-green discoloration of the nail plate was noted [4]. Such findings were idiosyncratic and not broadly generalized.

#### 3.3.9. Pooled Prevalences and Heterogeneity

Table 2 presents the pooled prevalence of key dermoscopic features along with heterogeneity metrics. In summary, localized hyperkeratosis and onycholysis were the most pervasive findings (present in >80% of lesions), followed by irregular/fuzzy borders (~75%) and splinter hemorrhages (~50%). Nail plate thickening occurred in ~45%, and polymorphic vessels (when observable) in ~90% after nail removal (though in only ~10% prior to removal). Longitudinal melanonychia was present in ~12% overall but in nearly all (89%) of the in situ cases. We observed considerable heterogeneity for some features (e.g., *I^2^* = 36% for splinter hemorrhages and *I^2^* = 48% for nail plate deformity), reflecting differences in patient populations and possibly reporting bias. The heterogeneity was partly explained by histologic subtype. In subgroup analyses, invasive SCC cases had significantly higher prevalence of hyperkeratosis and hemorrhages than in situ cases. Meanwhile, melanonychia was almost exclusive to the in situ subgroup, yielding near-complete separation. A meta-regression confirmed that the proportion of in situ lesions in a study was inversely associated with that study’s reported prevalence of hyperkeratosis and positively associated with the prevalence of melanonychia (*p* < 0.01 for both associations). This finding supports the notion that Bowen’s-type SCC and invasive SCC have somewhat divergent dermoscopic profiles, the former often pigmentary, the latter more keratotic. No significant effect of publication year on feature prevalence was found (*p* > 0.1), suggesting that the reporting of these dermoscopic signs has been relatively consistent over time. We noted that larger series (with >10 cases) tended to systematically assess certain features (like hyperkeratosis, onycholysis) in every case, whereas single-case reports sometimes focused on unique features of that case; this may have introduced some reporting bias in favor of unusual findings in the literature at large.

Furthermore, pooled diagnostic accuracy data revealed that several dermoscopic signs were strongly associated with subungual SCC when compared with benign mimickers. As shown in Table 3, a polymorphous vascular pattern yielded the highest diagnostic odds ratio (DOR = 12.6; 95% CI: 8.3–19.1; *p* < 0.001) when contrasted with viral warts. Localized subungual hyperkeratosis and dotted/glomerular vessels also demonstrated significant discriminative values (DORs = 6.8 and 9.3, respectively) relative to onychomycosis. Notably, non-parallel onycholytic borders were over 15 times more likely to be observed in SCC than in onychomatricoma (*p* < 0.001). These findings support the diagnostic utility of dermoscopy in distinguishing SCC from clinically similar but histologically benign nail disorders.

## 4. Discussion

In this systematic review and meta-analysis, we aggregated the dermoscopic characteristics of subungual squamous cell carcinoma from 121 published cases. Despite the rarity of this malignancy, our findings demonstrate that certain dermoscopic hallmarks consistently emerge, and knowledge of these can improve clinical suspicion and earlier diagnosis. The most prominent features, subungual hyperkeratosis, distal onycholysis, irregular lesion borders, and splinter hemorrhages, reflect the hybrid destructive and proliferative nature of nail unit SCC. These correspond well with what is known of SCC’s clinical behavior in the nail, it tends to produce a keratotic tumor that lifts the nail plate and often bleeds or oozes [38]. Notably, we found that dermoscopic hyperkeratosis and onycholysis were present in nearly all invasive SCCs, suggesting that any chronic onycholytic process with a persistent keratotic nodule underneath should raise a red flag for possible SCC. This is important because such cases are easily misdiagnosed as onychomycosis or verruca and treated ineffectively for months [39].

Several of the dermoscopic criteria identified in our review align with prior observations in smaller studies. Teysseire et al. (2017) had pointed out the significance of localized hyperkeratosis and the absence of well-defined edges in SCC [3]. Our meta-analysis reinforces their findings across a broader literature sample, hyperkeratotic onycholysis with irregular margins is a unifying theme in nail SCC. By contrast, onychomatricoma (a benign matrix tumor) typically shows a regular band of longitudinal perforations and parallel lesion edges on dermoscopy [3], features seldom seen in SCC. Likewise, verruca vulgaris of the nail bed can produce onycholysis and hyperkeratosis, but dermoscopically such lesions show dense thrombosed capillaries (black dots) in a pattern and lack the polycyclic invasive front of SCC [40]. In one comparative review, “oozing” (serosanguineous exudate) was noted as more typical for SCC than for verrucae [38] corresponding to the dermoscopically observed hemorrhagic crusts. Chronic onychomycosis can produce somewhat similar nail plate dystrophy and subungual debris, but usually fungal debris is more yellow-white and lacks the hemorrhagic spots and aberrant vascular patterns of SCC [39]. In addition, onychomycosis often affects multiple nails, whereas SCC is usually in one nail. However, differentiation can be very challenging, in fact, cases of SCC developing after longstanding “onychomycosis” have been reported (likely the SCC was present but assumed to be fungal) [41]. Dermoscopic examination may help identify the presence of blood spots, irregular termini of onycholysis, and frank tumorous structures (like a papillary mass) and should prompt a biopsy rather than another antifungal trial [38].

Our analysis also sheds light on the distinct dermoscopic profile of pigmented subungual SCC (Bowen’s disease). This entity is important primarily because it masquerades as melanoma. Several reports in our series involved patients who were initially suspected to have subungual melanoma due to a dark longitudinal streak, but biopsies revealed SCC in situ [18,29,30,31,32]. Dermoscopically, differentiating pigmented Bowen’s from melanoma is difficult because both can have irregular pigmentation and lack the clear linear patterns of benign melanonychia; therefore, in those cases biopsy remains mandatory. Nonetheless, some clues have been proposed, pigmented Bowen’s often has a grayer, more homogeneous pigmentation and may show glomerular vessels when examined closely or with the nail plate removed [18]. Melanoma of the nail matrix, on the other hand, classically exhibits the multicolored (brown, black, gray) longitudinal band (melanonychia striata), or other features like Hutchinson’s sign (periungual extension of pigment) [42]. None of the pigmented SCC cases in our review showed a clear longitudinal band; instead, they either showed diffuse brown color or an irregular pattern without parallelism, and notably, some had concurrent nail plate dystrophy or hyperkeratosis. This suggests that if a longitudinal melanonychia is accompanied by nail plate destruction or subungual keratin, one should consider Bowen’s disease. In practice, distinguishing a pigmented SCC in situ from a melanoma requires a biopsy and histopathology or adjunctive techniques like in vivo confocal microscopy. In one illustrative case, reflectance confocal microscopy of a pigmented nail streak revealed atypical keratinocyte clusters and pleomorphic nuclei (consistent with SCC in situ) rather than dendritic melanocytes [7]. Such advanced imaging may become more widely used to evaluate melanonychia and could assist in preoperative identification of SCC cases.

The vascular features seen on dermoscopy of nail SCC also parallel those known from cutaneous SCC and Bowen’s disease. Glomerular (dotted) vessels on a reddish background are a hallmark of Bowen’s disease on skin [36], and our review confirms that the same pattern occurs in the nail bed lesions once visible. Some authors have suggested that performing intraoperative dermoscopy (after removing the nail plate) should be a standard adjunct during Mohs surgery or excision of nail unit tumors [42]. By performing so, the surgeon can immediately inspect the surgical field dermoscopically for any residual tumor (which would appear as remaining glomerular vessels or keratinous foci) and extend resection until these signs are cleared [21]. While this practice is not yet widespread, it holds promise for micrographically controlling nail SCC excisions, especially in anatomically complex nail units where standard 5 mm margins are hard to apply. However, we noted that a recent study by Lee et al. examining pre- and post-avulsion dermoscopy did not find a reliable relationship between dermoscopic features and depth of invasion [37]. For example, the presence of a milky-red area or hairpin vessels did not consistently indicate a thicker tumor as one might expect [37]. Therefore, dermoscopy cannot substitute for histologic assessment of invasion (Breslow thickness), it is primarily a diagnostic aid and a tool for mapping tumor extent, not for gauging vertical depth. Intraoperative dermoscopy represents a promising adjunct; however, it requires prospective validation.

Several pooled estimates in this review exhibited moderate-to-substantial heterogeneity (*I*^2^), particularly for features that depend on fine vascular or margin detail (for example, irregular borders and polymorphous vessels). Multiple, partly overlapping factors likely drive this variability. First, histologic subtype (invasive versus in situ) materially alters lesion appearance and, in meta-regression, explained a significant proportion of between-study variance (studies with higher proportions of in situ lesions reported more pigmentary changes and less hyperkeratosis; *p* < 0.01). Second, the imaging context strongly influences visibility, whether imaging was performed with the nail plate intact or after nail removal markedly changes detection of vessels and margins (vessels are frequently only apparent after nail avulsion), and several studies did not report nail plate status. Third, heterogeneity is amplified by study design and reporting bias, small case reports preferentially publish atypical or striking presentations, whereas cohort studies more systematically report predefined features. Finally, variable operational definitions (what constitutes an “irregular” border or a “glomerular” vessel), differing case mixes (prior treatments, digit involved, immunosuppression/HPV status), and small sample sizes further increase between-study variability. Taken together, these factors suggest that pooled prevalences for features such as irregular borders should be interpreted with caution; future studies should use standardized feature definitions, prospectively record imaging parameters (including nail plate status), and deposit annotated image sets to reduce heterogeneity and improve the reliability of pooled estimates.

Clinically, the findings of this review have several implications. First and foremost, we advocate that dermoscopic examination be incorporated into the routine evaluation of any chronic nail unit abnormality of uncertain cause. Dermoscopy, as an inexpensive and noninvasive diagnostic procedure, may reduce unnecessary surgeries [43]. In the context of nail SCC, using dermoscopy could shorten the time to diagnosis by guiding timely biopsies, thereby potentially preventing the disease from advancing to bone invasion [23]. Another implication is patient education and follow-up patients with risk factors (e.g., solid organ transplant recipients with HPV warts, chronic nail trauma, etc.) should be educated that new persistent nail growths are not always benign. If a lesion is being observed or treated conservatively, close dermoscopic monitoring for changes (such as new hemorrhages, expansion of keratosis, or emergence of pigment) can be helpful.

From a research perspective, this systematic review highlights the need for more standardized and prospective studies on nail unit SCC. Almost all data we pooled were retrospective and subject to reporting biases. A prospective registry of nail tumors where dermoscopic features are systematically recorded would allow more accurate prevalence estimates and could help develop a diagnostic algorithm or scoring system. For instance, a combination of features (hyperkeratosis + hemorrhage + irregular border) might yield a high positive predictive value for SCC that could be formally studied. Additionally, future research could explore the dermoscopic differentiation between subungual SCC and its benign look-alikes. One interesting avenue is the use of dermoscopic algorithms or checklists similar to those used for skin tumors (e.g., the ABCD rule for melanoma) but modified for nails. A conceivable “ONYCHOSCC” checklist might include criteria like O: onycholysis (irregular), N: nail bed hyperkeratosis, Y: yellow-white opacity, C: coiled vessels, H: hypergranulation, O: oozing, S: Side-wall involvement/paronychia, C: Crumbling/destruction of plate, C: Chronic persistence despite therapy, to flag lesions that need biopsy. Such a system would need validation.

Another area for future investigation is the role of digital dermoscopy monitoring for high-risk patients. For example, transplant patients on long-term immunosuppression (who are prone to HPV-related SCCs) might benefit from periodic dermoscopic exams of nails, similar to how they obtain skin checks. Whether this could lead to earlier detection (and thereby smaller, more easily treated SCCs) is an open question. Moreover, given the overlaps between SCC in situ and melanoma in situ in nails, research into adjunctive technologies is warranted. Confocal microscopy, as demonstrated in some cases can identify nuclear atypia and architectural disarray in SCC in situ [28]. High-frequency ultrasound or optical coherence tomography (OCT) might also be applied to nail lesions to assess thickness and invasion, complementing dermoscopy [44,45]. Advances in artificial intelligence (AI)-assisted image analysis show promise to reduce operator dependence and help triage nail lesions, recent work has demonstrated feasibility of AI-assisted dermatologic screening for keratinocytic and benign lesions, including SCC [46]. Likewise, hyperspectral imaging (HSI) and other spectral modalities, especially when combined with AI, can detect tissue signatures beyond conventional color and morphology and may further improve discrimination of benign versus malignant nail lesions [46]. Prospective validation using standardized, device-annotated nail image sets is required before clinical implementation.

Our pooled estimates and diagnostic odds ratios are largely derived from case reports and small case series and therefore should be interpreted cautiously, the descriptive nature of many primary reports and the modest overall sample size increase the risk of overinterpretation and limit generalizability to broader clinical practice. Some studies contributed multiple lesions from the same center or patient, and although we pooled at the study level, within-study clustering was not consistently modeled; this non-independence can reduce the effective sample size and lead to overly narrow confidence intervals. We observed moderate heterogeneity (*I*^2^ values in the moderate range) for several features, which introduces uncertainty about the stability of pooled prevalences across settings and underscores the need for standardized reporting. Importantly, several vascular signs were substantially more frequent in images obtained after nail removal; these post-avulsion findings may not generalize to routine, pre-operative dermoscopic assessment and should not be taken as routinely observable in intact-nail exams. Where data permitted, we performed sensitivity checks and stratified analyses to explore these effects, but the limited and heterogeneous evidence base constrains definite conclusions. We acknowledge that DerSimonian–Laird estimation and the Freeman–Tukey transformation can be unsuitable when the number of studies is small or when event rates are very low or very high. Therefore, future studies consider alternative models (e.g., generalized linear mixed models, Bayesian approaches) for sensitivity analyses.

For this review, we defined subungual SCC as lesions originating within the nail bed or nail matrix of fingers or toes. We excluded studies that reported only periungual SCC (tumors limited to periungual skin without subungual involvement) because periungual and subungual tumors can differ in clinical presentation, dermoscopic appearance and pathogenesis; restricting to anatomically defined subungual lesions improves interpretability of pooled dermoscopic features. The exclusion of periungual SCC might represent a limitation because potentially informative adjacent-site lesions were not analyzed.

We also excluded articles lacking dermoscopic information, studies that described mixed/collision tumors without separable dermoscopic features attributable to SCC, conference abstracts without a full text, non-original reviews, and non-English language reports. We acknowledge that this restriction can introduce language bias by excluding potentially relevant non-English studies.

Moreover, data regarding epidemiology differ from already published datasets, probably because we focused only on the cases with available dermoscopy. Therefore, they should be approached with caution.

## 5. Conclusions

As discussed, the evidence mostly comprises uncontrolled observations. This inherently limits the strength of conclusions we can draw. We cannot, for instance, calculate the sensitivity or specificity of a dermoscopic feature for SCC based on these data, because comparative benign cases were not systematically studied in most reports. Future studies could address this by prospectively applying dermoscopy to all patients with said nail onycholysis and observing how many of those turn out to be SCC vs. other cases. Our review does not answer that kind of a question, but it lays a foundation by cataloging what features SCC tends to have.

Despite limitations, this review offers the most comprehensive synthesis to date of dermoscopic findings in subungual squamous cell carcinoma (121 cases). We found that features such as distal onycholysis with focal subungual hyperkeratosis, splinter hemorrhages, and diffuse nail bed erythema are common and should raise suspicion for SCC in otherwise indeterminable nail dystrophy. Dermoscopy might help to distinguish SCC from common mimics (warts, fungal infection), which rarely show the same combination of extensive keratin, polymorphic blood spots and invasive borders (but there is a need for further validation in comparative studies). It can identify warning signs (for example, associated keratosis or atypical vessels) that mandate biopsy; however, a tissue diagnosis remains essential. Clinically, we recommend dermoscopic evaluation of high-risk or persistent unexplained chronic nail lesions and prompt biopsy when one or more characteristic SCC signs are present, to allow earlier tissue-sparing treatment. Finally, better documentation of dermoscopic findings, comparative studies with benign conditions, and multimodal imaging research will strengthen diagnostic accuracy in the future.

## Figures and Tables

**Table 1:** Characteristics of included studies and patient demographics.

Article	Article Type	Lesion Number	Age	Sex	Localisation (Number of Lesions)
Serret CA et al. [19]	case report	1	62	M	III LF
Costa C et al. [20]	case series	3	60, ND, 53	F, ND, M	III RF, V RF, I LT
Göktay F et al. [21]	case report	1	55	M	I RF
Nojima K et al. [22]	case report	1	80	F	I RF
Ogata D et al. [23]	case report	1	46	M	IV LF
Massa AF et al. [24]	case report	1	47	M	I RT
Cinotti E et al. [25]	case report	1	60	f	IV LF
Mao DD et al. [26]	case report	1	57	M	IV LF
Gatica-Torres M et al. [27]	case report	1	20	M	II LF
Debarbieux S et al. [28]	case series	4	77, 86, ND, ND	M, F, ND, ND	ND, ND, ND, II RF
Fernández-Sánchez M et al. [29]	case-series	1	25	M	IV RF
Saito T et al. [30]	case report	1	72	M	I LF
Inokuma D et al. [31]	case report	1	41	M	II RF
Holanda GM et al. [18]	case report	1	64	F	III LF
Hou YL et al. [32]	case report	1	47	M	II RF
Williams NM et al. [33]	case series	2	38, 51	F, F	V RT, III RF
Sanchez-Carpintero I et al. [34]	case report	1	58	M	ND
Waterton K et al. [4]	case report	1	75	M	V RF
Carlioz V et al. [17]	monocentric retrospective cohort	53	30 to 80 years (mean 62)	31 F and 22 M	I LF (6), I RF (10), II LF (1), II RF (3), III RF (1), III LF (1), IV RF (1), V RF (2), V LF (2), I RT (8), I LT (14), II RT (1), III RT (1), IV RT (1), V RT (1)
Teysseire S et al. [3]	monocentric retrospective cohort	44	32 to 88 years (mean 61.77).	22 F and 22 M	I LF (6), I RF (11), II LF (2), III RF (1), IV LF (2), IV RF (1), V LF (2), I RT (8), I LT (7), II LT (1), III RT (1), IV RT (2)
**Total (pooled)**	—	121	**56.4 ± 16.3**	**60F/56M**	**Fingers 59.48%,** **Toes 40.52%**

Legend: F—female, LF—left finger, LT—left toe, M—male, ND—no data, RF—right finger, RT—right toe.

**Table 2:** Pooled prevalence of key dermoscopic features in subungual squamous cell carcinoma.

Dermoscopic Feature	Pooled Prevalence (95% CI)	*I*^2^ (%)
Polymorphous vascular pattern	87% (78–93)	64
Dotted/glomerular vessels	85% (73–92)	72
Irregular linear/serpentine vessels	74% (60–85)	68
Focal hyperkeratosis (whitish keratotic areas)	66% (53–78)	71
Non-parallel onycholytic borders	71% (55–85)	63
Hemorrhagic dots and streaks	22% (13–34)	59

**Table 3:** Diagnostic odds ratios (DOR) of key signs of squamous cell carcinoma versus benign mimics.

Dermoscopic Sign	Comparator	DOR (95% CI)	*p*-Value
Polymorphous vascular pattern	Verruca vulgaris	12.6 (8.3–19.1)	<0.001
Focal hyperkeratosis	Onychomycosis	6.8 (1.8–24.5)	0.02
Dotted/glomerular vessels	Onychomycosis	9.3 (4.1–21.3)	<0.001
Non-parallel onycholytic borders	Onychomatricoma	15.4 (5.0–47.5)	<0.001
Hemorrhagic structures	Nail hematoma	4.5 (1.2–16.7)	0.03

## Data Availability

The datasets generated during and/or analyzed during the current study are available from the corresponding author upon reasonable request.

## Supplementary Materials

The following supporting information can be downloaded at: https://www.mdpi.com/article/10.3390/cancers18030446/s1, Supplementary S1: The detailed search strategy.
