# Supplementary material for: Dermoscopy of Subungual Squamous Cell Carcinoma: A Systematic Review
Source: Cancers (Basel). 2026 Jan 30;18(3):446. doi: 10.3390/cancers18030446 (PMC12896984; doi:10.3390/cancers18030446)
Supplement: Supplementary file 1 [file cancers-18-00446-s001.zip › cancers-4099143-supplementary.pdf]

Supplementary S1: The detailed search strategy.

A comprehensive literature search was carried out in PubMed (MEDLINE), Scopus and the Cochrane Central Register of Controlled Trials (CENTRAL) from database inception through to 31 December 2023. Search strings were developed to capture terminology for squamous cell carcinoma of the nail unit and dermoscopic examination. The exact search strings were as follows:

- PubMed (MEDLINE) search string (used 31 December 2023):
  (("squamous cell carcinoma"[tiab] OR "SCC"[tiab] OR "Bowen"[tiab]) AND ("nail"[tiab] OR "subungual"[tiab] OR "nail unit"[tiab] OR "ungual"[tiab] OR "periungual"[tiab]) AND ("dermoscopy"[tiab] OR "dermatoscopy"[tiab] OR "onychoscopy"[tiab] OR "dermatoscope"[tiab]))

- Scopus search string (adapted for Scopus syntax, used 31 December 2023):
  TITLE-ABS-KEY(("squamous cell carcinoma" OR SCC OR Bowen) AND (nail OR subungual OR "nail unit" OR ungual OR periungual) AND (dermoscopy OR dermatoscopy OR onychoscopy OR dermatoscope))

- Cochrane CENTRAL search string (used 31 December 2023):
  ("squamous cell carcinoma" OR SCC OR Bowen) AND (nail OR subungual OR "nail unit" OR ungual OR periungual) AND (dermoscopy OR dermatoscopy OR onychoscopy OR dermatoscope)

Search strategies were adapted as required for each database's syntax. No date restrictions were applied, except for the final date included above. We additionally screened the reference lists of all included papers and relevant reviews (backward citation searching) and used forward citation tracking where helpful to identify additional eligible reports. After de-duplication, titles and abstracts were screened independently by two reviewers for potential eligibility.
